# Transcriptome Analysis Reveals the Flexibility of Cordycepin Network in *Cordyceps militaris* Activated by L-Alanine Addition

**DOI:** 10.3389/fmicb.2020.00577

**Published:** 2020-04-24

**Authors:** Bai-Xiong Chen, Tao Wei, Ling-Na Xue, Qian-Wang Zheng, Zhi-Wei Ye, Yuan Zou, Yi Yang, Fan Yun, Li-Qiong Guo, Jun-Fang Lin

**Affiliations:** ^1^Institute of Food Biotechnology & College of Food Science, South China Agricultural University, Guangzhou, China; ^2^Research Center for Micro-Ecological Agent Engineering and Technology of Guangdong Province, Guangzhou, China; ^3^Guangzhou Alchemy Biotechnology Co., Ltd., Guangzhou, China

**Keywords:** cordycepin, *Cordyceps militaris*, metabolic network, flexibility, transcription factor

## Abstract

Cordycepin, isolated from the traditional medicinal fungus *Cordyceps militaris*, has gained much attention due to its various clinical functions. Previous reports of L-alanine addition could significantly improve cordycepin production, but the molecular mechanism remains unknown. In this study, transcriptome analysis of *C. militaris* with doubled cordycepin production induced by L-alanine addition provides an insight into the flexibility of the cordycepin network. The biopathways of energy generation and amino acid conversion were activated so that cordycepin substrate generation was consequently improved. Specific genes of rate-limiting enzymes in these pathways, as well as related transcription factors, were figured out. Two key Zn2Cys6-type transcription factors CmTf1 and CmTf2 were verified to play the roles of doubling the cordycepin production by overexpression of their coding genes in *C. militaris* wild type. These results provide a complete map of the cordycepin network in *C. militaris* with a distinct understanding of the flexibility of joints, giving a better foundation for increasing cordycepin yield and strain breeding in the future.

## Introduction

*Cordyceps militaris* has been popular in East Asia for centuries as a kind of edible and medicinal fungus. Recently, the application of various bioactive metabolites isolated from *C. militaris* led to rapid development in food, pharmaceutical, and cosmetics industries (Paterson, 2008; Lee et al., 2009; Zhou et al., 2009). To extend the applications of *C. militaris*, its bioactive metabolites, such as adenosine, ergosterol, ergothioneine (Cohen et al., 2014), *Cordyceps* polysaccharides (Zhang et al., 2015), and cordycepin (Cunningham et al., 1950; Paterson, 2008), are extracted and made into tablets and capsules. Among these biologically active ingredients, cordycepin has attracted the most attention for its multiple therapeutic effects (Tuli et al., 2014). Cordycepin (3′-deoxyadenosine) was proved to be able to inhibit cell proliferation and induce cell apoptosis (Tian et al., 2015) by binding signaling molecules, which led to anti-inflammatory effect (Noh et al., 2008). By preventing excessive glutamate-induced oxidation and endoplasmic reticulum stress-associated apoptosis in hippocampal cells (Jin et al., 2014), it was also proved to have antioxidant effects (Kim et al., 2006). Besides, cordycepin was proved to have broad-spectrum antibiotic activity by inhibiting NAD^+^-dependent DNA ligase (Zhou et al., 2016).

To optimize the production of this medical component, various kinds of energy sources (Mao et al., 2005; Mao and Zhong, 2006), putative precursors (Gu et al., 2007), mineral salts (Le et al., 2006; Fan et al., 2012), and culture conditions (Masuda et al., 2007; Kang et al., 2014) in *C. militaris* culturing had been tested. The highest production of cordycepin was reported by adding L-alanine in liquid static *C. militaris* fermentation, yielding up to 2 g/L (Kang et al., 2014). However, a detailed mechanism at the genetic level is still rarely reported.

High-throughput sequencing technologies have been used to discover the biosynthetic and metabolic pathway of cordycepin (Zheng et al., 2011; Yin et al., 2012; Raethong et al., 2018). As an isomer of 2′-deoxyadenosine, cordycepin was predicted to share a similar synthesis pathway of 2′-deoxyadenosine, but the relevant key enzyme was missing in the genome of *C. militaris* (Zheng et al., 2011). This revealed that cordycepin might have a different metabolic pathway. The cordycepin biosynthetic pathway was narrowed in the purine metabolism pathway by transcriptome and proteome analysis of two different stages of *C. militaris* (Yin et al., 2012). A three-gene biosynthesis cluster was reported (Xia et al., 2017) to synthesize cordycepin from adenosine, and it revealed that cordycepin metabolism overlapped with parts of the purine pathway. Further transcriptome analysis of samples with three carbon sources (Raethong et al., 2018) had tried to draw a network between adenosine, methionine, and cordycepin. Unfortunately, it failed to provide further verification in a molecular level.

To better understand the molecular mechanism of L-alanine’s action on cordycepin production, a transcriptome analysis was carried out in this study. The transcription data revealed key rate-limiting enzymes and transcription factors with high flexibility in cordycepin synthesis, and a metabolic network map from the substrate amino acid to the product cordycepin in *C. militaris* was drawn. In this study, two key Zn2Cys6-type transcription factors CmTf1 and CmTf2, which were highly transcribed after the adding of L-alanine, were overexpressed and verified to be the function of improving the cordycepin yield *in vivo*. Improved production of cordycepin in CmTf1/2 overexpressed strains reveal that the Zn2Cys6-type transcription factors (TFs) were not only involved in the development of *C. militaris* fruiting (Zheng et al., 2011) but also joined in the regulation of secondary metabolites in *C. militaris*.

## Materials and Methods

### Culture Conditions

**FIGURE 1:**
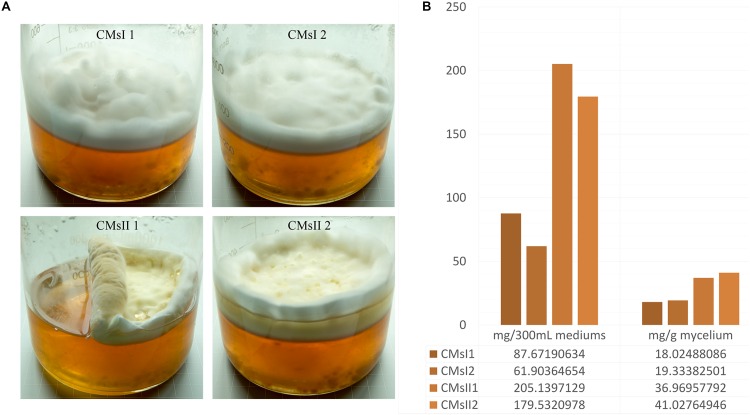
Characteristics of the four studied samples. **(A)** Growth characteristics of the four samples in glass jars after 30 days of culturing. **(B)** Cordycepin production in each glass jar.

Two types of media were modified from a previous report (Kang et al., 2014). A basal medium, containing (g/L) peptone 20, sucrose 24.7, K_2_HPO_4_⋅3H_2_O 1.11, MgSO_4_⋅7H_2_O 0.90, and Vitamin B_1_ 0.01, was used and 8 g/L L-alanine was added as treatment. *C. militaris* was incubated on Potato Dextrose Agar slants at 25°C for 7 days to form mycelium pellets. The pellets were transferred into a 250 ml flask contained 70 ml basal medium and cultured at 150 rpm min^–1^ for 8 days to expand the culture. The liquid static fermentation was performed in a 1-L wide-mouth glass jar (Figure 1A), 30 ml mature *C. militaris* CM10 mycelium pellets mixed with 270 ml of basal medium or its treatment, to build the samples of CMsI or CMsII. The jars maintained stationary in the dark at 25°C for 30 days. Mycelium layer on the surface from the sample was collected, frozen, and dried.

Since the static fermentation of *C. militaris* needs to take a longer time and harder to make samples equal, the shake-flask fermentation was performed to verify the impact of in *vivo* TF overexpression on cordycepin production. The mycelium of *C. militaris* CM10Tf1 and CM10Tf2 was collected from fresh plates and inoculated into 100 ml of PPDB (potato extracts 200 g/L, glucose 20 g/L, MgSO_4_⋅7H_2_O 1.5 g/L, KH_2_PO_4_ 3 g/L, peptone 10 g/L, VB1 10 mg/L) medium. After fermentation at 25°C with 150 rpm for 5 days, the medium was supplemented with 8 g/L L-alanine and continued to shake for 3 days before the harvest.

### HPLC Detection of Cordycepin

After fermentation, the mycelia were lyophilized and ground to powder. Two grams of dry powder was weighted from each sample to perform HPLC detection. HPLC assay was performed on Model LC2000 Liquid Chromatography System (TECHCOMP, Shanghai, China) equipped with an ULTIMATE AQ-C18 HPLC Column (4.6 × 250 mm, 5-Micron; Welch, Shanghai, China). The analysis conditions were as follows: mobile phase, 85% ultra-pure water and 15% methanol (v/v); flow rate, 1 ml/min; detection wavelength, 260 nm; injection volume, 20 μl. A standard cordycepin curve was generated using 0.02–0.25 μg/ml cordycepin standard (Sigma-Aldrich, United States). The cordycepin yield was calculated using the detected peak area according to the standard curve. The cordycepin concentration of mycelia presented in the study was calculated by normalizing in the equal biomass.

### Extraction of Total RNA, Library Establishment, and Transcriptome Sequencing

Total RNA was extracted from 100 mg of frozen mycelium pellets using the E.Z.N.A. Fungal RNA Miniprep Kit (OMEGA Bio-Tek, United States). After DNase I treatment, the quality of RNA was assessed by a NanoDrop and Agilent 2100. Oligo(dT) was used to isolate mRNA. The mRNAs were fragmented and used as templates to synthesize cDNA. Short fragments were purified and processed by end repair, single-nucleotide adenine addition, and adapter connection. Suitable fragments were selected for PCR amplification. The quantification and qualification of the sample library were performed by an Agilent 2100 Bioanalyzer (Agilent, Beijing, China) and an ABI StepOnePlus Real-Time PCR System (Thermo Fisher Scientific, United States), and further analyzed by following the operating instruction. The library was sequenced using the Illumina HiSeq by BGI (Shenzhen, China).

### Mapping the RNA-Seq Reads and Quantitative Analysis of the Genes

After sequencing, to obtain clean reads, raw reads were filtered for low-quality (the percentage of base whose quality is lesser than 15 is greater than 20% in a read), adaptor-polluted reads, and reads with a high content of unknown bases (unknown bases are more than 5%) using BGI (Shenzhen, China) in-house software with the quality system phred64 were removed and clean reads were stored in FASTQ (Cock et al., 2010) format. Clean reads were uploaded to NCBI (Submission ID: PRJNA413637) and mapped to the reference genome *C. militaris* CM01 (NCBI accession number. AEVU00000000) using HISAT (version: v0.1.6-beta; parameters: –phred64 –sensitive –no-discordant –no-mixed -I 1 -X 1000) (Kim et al., 2015). StringTie (Pertea et al., 2015) (version: v1.0.4; parameters: -f0.3 -j3 -c 5 -g100 -s 10000 -p8) and CuffCompare (Trapnell et al., 2012) (version: v2.2.1; parameters: -p12) were used to reconstruct the transcripts and compare with the reference annotation, respectively. CPC (Kong et al., 2007) (version: v0.9-r2; parameters: default) software was further used to predict the potential novel coding transcripts. These predicted transcripts were merged with the reference transcripts to obtain a complete reference. For gene expression analysis, clean reads were mapped to the complete reference using Bowtie2 (Langmead and Salzberg, 2012) (version: v2.2.5; parameters: -q –phred64 –sensitive –dpad0 –gbar99999999 –mp1, 1 –np1 –score -minL, 0, -0.1 -I1 -X 1000 –no-mixed –no-discordant -p1 -k 200) and analyzed with RSEM (Li and Dewey, 2011) (version: v1.2.12; parameters: default), which could be used to estimate gene and isoform expression levels from RNA-Seq data, to obtain the gene expression level for each sample. The gene expression level was determined by calculating the number of fragments in each sample and normalized to FPKM (Fragments Per Kilo-base of exon model per Million mapped reads).

### GO Functional Classification and KEGG Pathway Enrichment Analysis of DEGs

Differentially expressed genes (DEGs) were detected using edgeR criteria in R (Robinson et al., 2010) according to the RSEM results. The detection of DEGs between two treatments (CMsI vs. CMsII) was based on the noisy distribution method (NOIseq) (Tarazona et al., 2011), and the corrected *P* > 0.8 and absolute value of fold change > 2 were considered as DEGs, while detection of DEGs between every single sample (CMsI1 vs. CMsI2 vs. CMsII1 vs. CMsII2) was based on the Poisson distribution method (PoissonDis) (Audic and Claverie, 1997), and the false discovery rate, FDR < 0.001, and the absolute value of fold change > 2 were considered as DEGs. To further analyze the DEG annotations, Gene Ontology (GO) functional classification and a Kyoto Encyclopedia of Genes and Genomes (KEGG) pathway enrichment analysis were carried out based on the GO database (Ashburner et al., 2000) and KEGG database (Kanehisa et al., 2007).

### Quantitative Real-Time PCR Validations of DEGs

The qRT-PCR template cDNA was synthesized from 0.5 μg of total RNA by ReverTra Ace qPCR RT Master Mix (Toyobo, Osaka, Japan). All primers used for the quantitative real-time PCR (qRT-PCR) are listed in Supplementary Table S1. Each qRT-PCR reaction system had a total volume of 20 μl, containing 50 ng of cDNA, 160 nM relevant primers, and the SYBR Green Realtime PCR Master Mix (Toyobo). All qRT-PCR reactions were carried out in the CFX96 Real-Time PCR Detection System (Bio-Rad, CA, United States), following the reaction parameters in the instruction book from the SYBR Green Realtime PCR Master Mix (Toyobo, Osaka, Japan). Using the elongation factor 1-alpha (*tef1*) gene (NW_006271969.1) as an internal control for each sample (Lian et al., 2014), the relative gene expression levels were calculated by the 2^–ΔΔ^ CT method (Livak and Schmittgen, 2001).

### Overexpression of Putative Transcription Factors

The transcription factors CCM_02568 gene (accession number NW_006271970.1) and CCM_01481 (accession number NW_006271969.1) gene were cloned from *C. militaris* CM10 by primers Tf1F/Tf1R and Tf2F/Tf2R (Supplementary Table S10). These genes were renamed as *cmtf1/cmtf2* (represented the full-length gene of CCM_02568/CCM_01481, respectively) and constructed into plasmid p390-blpR-sgRNA-cmcas9-gfp (Chen et al., 2018) by *Xba*I/*Bcu*I using a ClonExpress II One Step Cloning Kit (Vazyme Biotech Co., Ltd, Nanjing, China) to build p390-blpR-CmTf1/2. Then the overexpressed vectors were transformed into *Agrobacterium tumefaciens* AGL-1 (Weidi Bio, Shanghai, China) by electroporation. The *A. tumefaciens*-mediated transformation (ATMT) was used to build the CmTf1/2 overexpression *C. militaris* as described in a previous study (Chen et al., 2018).

## Results

### Growth Characteristics and Cordycepin Production From *C. militaris* After L-Alanine Addition

After 30 days of liquid culturing, mycelia and the color of residual medium did not present any significant or sudden change, indicating that large-scale cell autolysis did not occur in the fermentation, and the amount of carbon source and nitrogen source in the medium was enough for the long period of mycelia growth. In static liquid culture, compared to the control, primordium in the CMsII group was visually thicker (Figure 1A). According to the standard curve formula, *y* = 1e-08*x*+0.001 (*R*^2^ = 0.9994), the cordycepin extraction yield detected by HPLC was obtained. The total amount of 36.9 and 41.0 mg/g of cordycepin was extracted from the dry mycelia harvested from the CMsI group, while the other group was 18.0 and 19.3 mg/g (Figure 1B). After adding L-alanine in the static liquid culture, the cordycepin concentration increased 2.1 times.

### Sequencing Data Analysis and Annotation

Four cDNA samples from *C. militaris* mycelia were processed by the Illumina HiSeq platform and, on average, generated 4.44 Gb of clean bases in each sample (Supplementary Table S2). After mapping the sequenced reads to the reference genome, on average 80.3% of the reads were mapped (Supplementary Table S3). After reconstruction, 4573 novel transcripts were predicted in all four samples. Among these, 4013 transcripts were previously unknown splicing events for known genes, while 68 were novel coding transcripts without any known features, and the remaining 492 were long non-coding RNAs.

**FIGURE 2:**
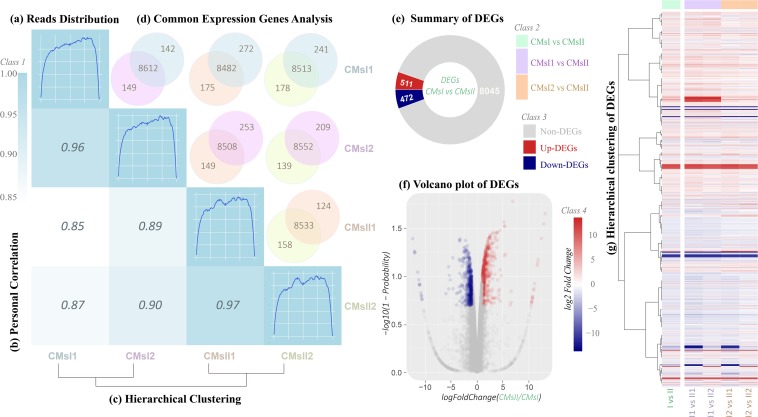
Total gene expression analysis. **(A)** Rough graph of read distribution (diagonal, details are shown in Supplementary Figure S1). **(B)** Heatmap of the Pearson correlations between samples (left, the *x-* and *y*-axis represented each sample. The coloring indicates the Pearson correlation, high: cyan, low: white). **(C)** Hierarchical clustering of samples (below, a closer distance indicates a more similar expression profile of samples). **(D)** Venn diagram of the commonly expressed genes between samples (above, the coloring indicates the sample identity: cyan, CMsI1; purple, CMsI2; orange, CMsII1; yellow, CMsII2). **(E)** Summary of the DEGs (CMsI vs. CMsII). **(F)** Volcano plot of the DEGs (CMsI vs. CMsII) (the *x*-axis represents the log2-transformed fold change. The *y*-axis represents the -log10-transformed significance) (coloring indicates the fold change: up-regulated DEGs, red; down-regulated DEGs, navy blue; non-DEGs, gray). **(G)** Heatmap of the hierarchical clustering of the DEGs (the *x*-axis represents each compared sample; the *y*-axis represents the DEGs. The coloring indicates the fold change: high, red; low, navy blue).

After the novel coding transcripts were merged with the reference transcript to obtain a complete reference, clean reads were mapped to this reference (Supplementary Table S4) to calculate the gene expression level for each sample. Based on the mapping results, the read distributions, the Pearson correlation coefficient between expression values across samples and hierarchical clustering between four samples were calculated (Figures 2A–C and Supplementary Figure S1).

### DEGs in *C. militaris*

Global transcriptional changes from normalizing the DEG data were analyzed to identify DEGs (Figure 2D). Compared to the treatment without the addition of L-alanine, there were 511 genes up-regulated and 472 genes down-regulated in the treatment (CMsI vs. CMsII) (Figure 2E). A volcano plot was drawn to show the global fold changes between the up-regulated and down-regulated genes (Figure 2F). As shown in the volcano plot, most of the DEGs represented a slight fold change, while some of the DEGs displayed significant differences. Results with this pattern revealed that adding L-alanine may regulate some unknown genes in the cordycepin metabolic network. Additionally, a heatmap was drawn to present the hierarchical clustering of the DEGs among samples (Figure 2G). As shown in the heatmap, the expression profiles between the replicates were similar, indicating that the transcriptome data were reliable.

### GO Analysis and KEGG Pathway Enrichment of DEGs

**FIGURE 3:**
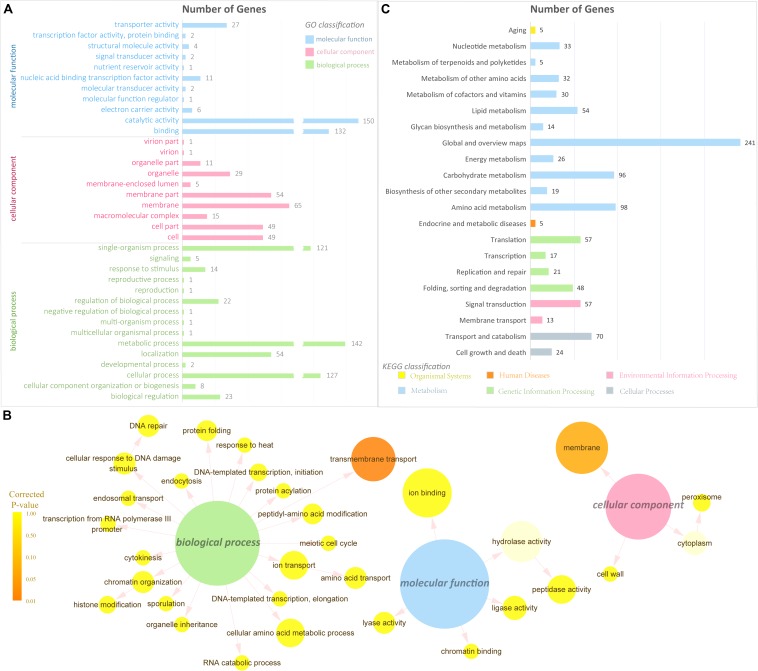
Gene Ontology and pathway analysis. **(A)** GO classification of the DEGs (the *x*-axis represents the number of DEGs, and the *y*-axis represents the GO term). **(B)** GO enrichment of the DEGs. The color of the column indicates the corrected *P-*value: (high, yellow; low, orange) or the ontology (blue, molecular function; pink, cellular component; green, biological process). **(C)** Pathway classification of the DEGs (the *x*-axis represents the number of DEGs, and the *y*-axis represents the pathway name).

GO classification and functional enrichment were performed for the gene annotation. A total of 523, 338, and 279 DEGs were assigned to the ontologies “biological process,” “molecular function,” and “cellular component,” respectively (Figure 3A). Over 100 DEGs were classified into five GO terms, including “cellular process,” “metabolic process,” “single-organism process,” “binding,” and “catalytic activity.” It indicated that a certain amount of DEGs was predicted to be correlated with transmembrane transport and signal transduction in the GO database. To further verify the GO ontologies results, a GO functional enrichment of the DEGs was performed and displayed by software Cytoscape (Shannon et al., 2003) with package BiNGO (Maere et al., 2005; Figure 3B). Terms in Figure 3B showing the smallest corrected *P-*value indicated that the Biological Process of transmembrane transport was significantly enriched in the DEGs (*P* < 0.01), suggesting its importance in the metabolism of supplementing L-alanine.

To have a better understanding of the interactions of functional genes by pathway-based analysis, all the genes were mapped to the KEGG database to categorize the biological functions of the DEGs. Specific gene enrichment was observed in 118 pathways. Twenty-one pathways were showing DEG enrichment (965 genes), and the cluster “Global and overview maps” was the largest group (241 genes, 25.0%). Clusters for “Amino acid metabolism” (98 genes, 10.2%), “Carbohydrate metabolism” (96 genes, 9.9%), “Transport and catabolism” (70 genes, 7.3%), “Signal transduction” (57 genes, 6.0%), and “Translation” (57 genes, 6.0%) were followed (Figure 3C). Moreover, the *P-*values of 12 pathways were less than 0.05 (Table 1). The pathways “Glycine, serine and threonine metabolism,” “Metabolic pathways,” and “Starch and sucrose metabolism” showed an even greater significant enrichment (*P* < 0.01).

**TABLE 1:** The smallest *P*-value (*P* < 0.05) pathway in KEGG.

	Pathway	DEGs	All genes	*P-*value	Pathways ID	Level 1	Level 2
1	Glycine, serine, and threonine metabolism	30	175	0.002566724	ko00260	Metabolism	Amino acid metabolism
2	Metabolic pathways	230	1962	0.003202936	ko01100	Metabolism	Global and overview maps
3	Starch and sucrose metabolism	19	104	0.007530212	ko00500	Metabolism	Carbohydrate metabolism
4	Aflatoxin biosynthesis	7	28	0.01876196	ko00254	Metabolism	Biosynthesis of other secondary metabolites
5	Synthesis and degradation of ketone bodies	4	11	0.0191878	ko00072	Metabolism	Lipid metabolism
6	Biosynthesis of antibiotics	67	523	0.02257833	ko01130	Metabolism	Global and overview maps
7	Taurine and hypotaurine metabolism	6	24	0.02885319	ko00430	Metabolism	Metabolism of other amino acids
8	ABC transporters	13	73	0.029823	ko02010	Environmental Information Processing	Membrane transport
9	Alanine, aspartate, and glutamate metabolism	16	98	0.03603956	ko00250	Metabolism	Amino acid metabolism
10	Staurosporine biosynthesis	7	32	0.03749299	ko00404	Metabolism	Biosynthesis of other secondary metabolites
11	Inositol phosphate metabolism	12	69	0.04231212	ko00562	Metabolism	Carbohydrate metabolism
12	Biosynthesis of amino acids	29	208	0.04533197	ko01230	Metabolism	Global and overview maps

**FIGURE 4:**
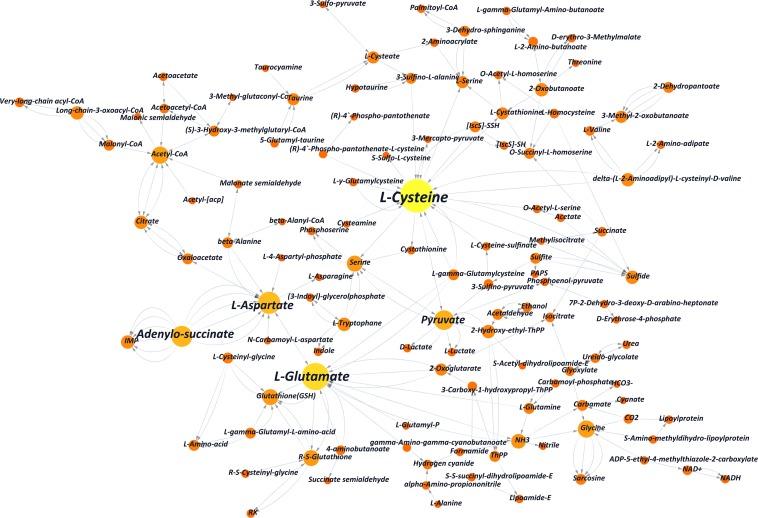
Partial substance transformation networks induced by L-alanine (the orange and yellow columns indicate metabolic substances involved in L-alanine metabolism; each red line indicates every single gene, which directs the related reaction, and the arrow indicates the direction of the transformation).

DEGs that took part in the pathway of autophagy regulation (KEGG pathway ko04140) were not shown enrichment, indicating that large-scale cell starvation did not occur in the fermentation. It also indicated that the increased yield of cordycepin was caused by the interaction affection of L-alanine in the metabolic network. A network map of substance interactions affected by L-alanine addition was obtained by the comparison between the transcriptome data and the KEGG database (Figure 4 and Supplementary Table S5). Interactions between substances were directed by the DEGs and eventually combined to draw one transformation network.

As shown in the network, L-cysteine and L-glutamate played key roles in substance transformation after adding L-alanine. Other amino acids, such as glutamine, glycine, and L-aspartate, which played an important role in this network had been reported to be involved in the purine pathway (Zheng et al., 2011). It indicated that additives like L-alanine may improve cordycepin production by interfering with amino acid conversion. Also, according to the comparison with KEGG pathway ko01100, significant transcriptional changes of genes were enriched in the pathways of the glycolysis of carbohydrate and amino acid metabolism, pentose phosphate pathway and citrate cycle of energy metabolism, the fatty acid pathway, and ribonucleotide metabolism (Supplementary Figure S2 and Supplementary Table S6).

### Putative Transcription Factors Involved in Amino Acid Metabolism and Production of Cordycepin

To further analyze the molecular interaction mechanism between L-alanine addition and cordycepin overproduction, the amino acid sequence of DEGs between CMsI and CMsII were uploaded into the Fungal Transcription Factor Database (Park et al., 2008) (FIFD)^1^ to detect putative transcription factors. Output candidates were further verified by InterPro^2^. A total of 94 putative TFs from 983 DEGs (10.5%) was predicted and classified into different TF families. These TF families contained Zn2Cys6 (MacPherson et al., 2006), HMG (Hall et al., 2006), Homeodomain-like (Kim et al., 2009), OB-fold nucleic acid binding (Taylor et al., 1995), C2H2 Zinc finger (Kim et al., 2009), Helix-turn-helix (Gallegos et al., 1997), bZIP (Dementhon et al., 2004), Winged helix repressor DNA-binding (Gajiwala and Burley, 2000), etc. (Table 2 and Supplementary Table S7).

**TABLE 2:** Transcription factors of DEGs from FTFD.

Transcription factors families	Determined	Undetermined	Total
APSES		1	1
Heteromeric CCAAT factors	1		1
NDT80/PhoG like DNA-binding	1		1
Negative transcriptional regulator	1		1
YL1 nuclear protein	1		1
AT-rich interaction region	1		1
Bromodomain transcription factor	1		1
p53-like transcription factor	1		1
Centromere protein B, DNA-binding region	1		1
Zinc finger, DHHC-type	1		1
Forkhead		1	1
bHLH	2		2
GATA type zinc finger	1	1	2
Homeobox	2		2
Bacterial regulatory protein		2	2
Tubby transcription factors	1	1	2
Myb	2		2
Lambda repressor-like, DNA-binding	1	1	2
Zinc finger, CCHC-type	2		2
Winged helix repressor DNA-binding	1	4	5
bZIP	6		6
Helix-turn-helix	5	1	6
C2H2 zinc finger	6	2	8
Nucleic acid-binding, OB-fold	7	1	8
Homeodomain-like	8	2	10
HMG	12	3	15
Zn2Cys6	20	1	21

The largest family was Zn2Cys6, followed by HMG and Homeodomain-like families. The significantly up-regulated TFs were the Zn2Cys6-type genes CCM_02568 (transcription level, CMsI: 1040.0, CMsII: 2750.2, 1.4-fold increased) and CCM_01481 (CMsI: 255.8, CMsII: 583.3, 1.2-fold increased). A previous work reported (Zheng et al., 2011) that the Zn2Cys6 TF genes CCM_01809 and CCM_09644 were relevant to fruit body formation; nevertheless, the Zn2Cys6-type TFs might also play an important role in the amino acid metabolism or cordycepin production.

### Putative Cordycepin Metabolism Pathway

**FIGURE 5:**
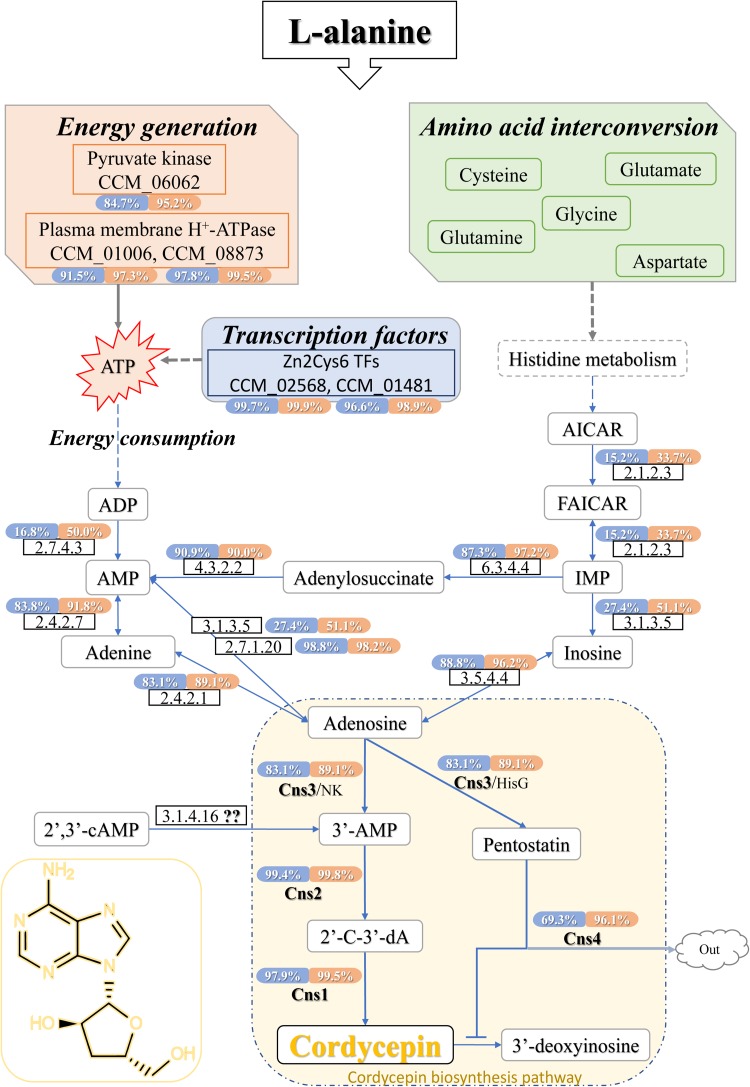
Cordycepin metabolism and biosynthesis pathway in *C. militaris.* The percentage showed in coloring indicates the ratio that the transcription level of this gene was higher than how many genes of DEGs in samples (CMsI, blue; CMsII, orange). The dotted lines indicate the indirect effects. The solid arrows indicate direct action. TFs, transcription factors; AICAR, 5-aminoimidazole-4-carboxamide ribonucleotide; FAICAR, 5-formamidoimidazole-4-carboxamide ribotide; IMP, inosine monophosphate; ATP, adenosine triphosphate; ADP, adenosine diphosphate; AMP, adenosine monophosphate; 2′,3′-cAMP, 2′,3′-cyclic monophosphate; 3′-AMP, adenosine-3′-phosphate; 2′-C-3′-dA, 2′-carbonyl-3′-deoxyadenosine.

Since the cordycepin production was significantly improved by L-alanine addition, DEGs related to cordycepin synthesis were carefully identified by comparison to the previously reported purine metabolic pathway constructed by Zheng et al. (2011), and Vongsangnak et al. (2017), and the cordycepin biosynthesis gene cassette presented by Xia et al. (2017). Finally, the whole cordycepin metabolic network in *C. militaris* was predicted (Figure 5). The detailed decipher of hypothetical genes (Table 3) involved in the network was as follows.

**TABLE 3:** Genes involved in putative cordycepin metabolic network.

Enzymes	EC number	Gene ID (Transcriptional level CMsII/CMsI, log2FoldChange (CMsII/CMsI))
Adenylosuccinate synthase	6.3.4.4	CCM_06768(315.3/102.4, 1.62)^a^, CCM_07353(2.7/1.8, 0.60)
Adenylosuccinate lyase	4.3.2.2	CCM_05789(122.1/130.0, -0.10)
Pyruvate kinase	2.7.1.40	CCM_06062(228.9/89.6, 1.35)^a^
Adenylate kinase	2.7.4.3	CCM_01353(33.1/42.8, -0.37), CCM_02335(34.2/33.4, 0.04), CCM_03940(25.4/6.1, 2.07)^a^, CCM_04983(31.7/59.1, 0.81), CCM_07479(48.2/38.3, 0.33)
Adenosine kinase	2.7.1.20	CCM_02717(446.2/493.0, -0.14)
Adenosine deaminase	3.5.4.4	CCM_02911(17.5/10.5, 0.74), CCM_07169(64.6/53.9, 0.43), CCM_07799(263.8/113.8, 1.21)^a^, CCM_09449(60.0/63.1, -0.07)
Adenine phosphoribosyltransferase	2.4.2.7	CCM_00088(145.2/85.9, 0.76), CCM_02051(0.78/0.82, -0.08)
5′-Nucleotidase	3.1.3.5	CCM_00622(26.2/11.9, 1.14), CCM_02830(0.27/0.89, -1.72), CCM_02831(11.8/10.4, 0.18), CCM_04038(3.2/0.01, 8.31), CCM_04644(31.6/23.5, 0.43), CCM_07972(1.2/11.6, -3.27)
Purine-nucleoside phosphorylase	2.4.2.1	CCM_04505(25.3/13.1, 0.94), CCM_04506(113.0/83.3, 0.44)
IMP cyclohydrolase	2.1.2.3	CCM_07593(13.8/5.2, 1.40)
Cordycepin synthetase 1	Cns1	CCM_04436(958.7/338.3, 1.50)^a^
Cordycepin synthetase 2	Cns2	CCM_04437(2194.3/709.0, 1.63)^a^
Cordycepin synthetase 3	Cns3	CCM_04438(238.4/143.5, 0.73)
Cordycepin synthetase 4	Cns4	CCM_04439(260.6/50.6, 2.36)^a^
H^+^-transporting ATPase	3.6.3.14	CCM_01006(323.5/136.4, 1.25)^a^
H^+^-transporting ATPase	3.6.3.6	CCM_08873(975.5/333.0, 1.55)^a^

*^a^Up-regulated DEGs.*

To begin with the left side of the network (Figure 5), a previous study (Mao and Zhong, 2006) showed that once the expression of plasma membrane H^+^-ATPase is suppressed, cordycepin production decreases, and ATP accumulates. The genes CCM_01006 (CMsI: 136.4, CMsII: 323.5) and CCM_08873 (CMsI: 333.0, CMsII: 975.5), annotated as plasma membrane H^+^-ATPase, were significantly up-regulated, which matched those of the previous report. CCM_06062, the significantly up-regulated putative DEG in *C. militaris*, was annotated as pyruvate kinase (classified as EC 2.7.1.40) according to sequence homology analysis. Its highly up-regulated transcriptional level will lead to the function of improving ATP generation. Since adenine could be obtained by the over-dephosphorylation of ATP, CCM_06062 indirectly increased the production of adenine, which is a plausible candidate substance of cordycepin. Other than adenine, AMP was also a plausible candidate substrate of cordycepin because previous report showed that adenylate kinase was a key enzyme in cordycepin synthesis (Kuo et al., 2015). Five genes were annotated as adenylate kinase (classified as EC 2.7.4.3) in this study. The CCM_03940 was the only significantly up-regulated one at the transcriptional level. CCM_01353, identified as a unique adenylate kinase gene in *C. militaris* in a previous report (Vongsangnak et al., 2017), along with the other three genes, both showed no significant difference between the experimental group and control. Another enzyme verified (Kuo et al., 2015) to be involved in cordycepin synthesis was adenine phosphoribosyltransferase (APRTase) (EC 2.4.2.7), which transforms adenine into AMP. The transcriptional level of its annotated gene CCM_00088 was 145.2 in the treatment group and 85.9 in the control, which was only 69% up-regulated in this study.

From the right side of the network (Figure 5), histidine metabolism was predicted to be involved because it is a bypass pathway of adenine formation. The transcriptional level of DEG CCM_06768, which was annotated as denylosuccinate synthase (EC 6.3.4.4) and could transform IMP into adenylosuccinate, was significantly up-regulated (CMsI: 102.4, CMsII: 315.3, 1.6-fold). For its downstream reaction, the gene CCM_05789 (the expressed enzyme classified as EC 4.3.2.2), for AMP formation from adenylosuccinate, showed no transcriptional difference between samples. However, its transcriptional level was higher than that of 90% of the DEGs (CMsI: 130.9, CMsII: 122.1). The gene CCM_02717, annotated as adenosine kinase (EC 2.7.1.20) and takes part in the transformation from AMP to adenosine, was similar to the gene CCM_05789. Its transcriptional level was also relatively high (CMsI: 492.9, CMsII: 446.2, higher than 99.98% of the DEGs), while no obvious difference was found between samples.

As followed, four genes were annotated as adenosine deaminase (EC 3.5.4.4), while only one DEG, CCM_07799 (CMsI: 113.8, CMsII: 263.8, 1.2-fold), was significantly up-regulated in the transcriptome data. A previous study speculated that the synthesis location for cordycepin and purine is separated (Lennon and Suhadolnik, 1976). The up-regulation of CCM_07799 indicated that adenosine deaminase may improve cordycepin production by converting adenosine into inosine in the pathway of cytosol RNA editing.

Finally, the network connected to the cordycepin biosynthesis cluster. The transcriptional levels of CCM_04436, annotated as *cns1* (CMsI: 338.3, CMsII: 958.7, 2.8-fold), and CCM_04437, annotated as *cns2* (CMsI: 709.0, CMsII: 2194.3, 3.1-fold), were both significantly up-regulated at similar levels. However, the transcriptional level of the gene CCM_04438, annotated as *cns3* (CMsI: 143.5, CMsII: 238.4, 1.7-fold), was only slightly increased. 3′-AMP could be also produced by phosphoesterase (EC 3.1.4.16) from 2′,3′-cAMP, which was generated during mRNA degradation. Though the phosphoesterase gene was not determined in *C. militaris*, 2′,3′-cAMP has been verified to be involved in cordycepin production (Xia et al., 2017).

Besides, 40 of the DEGs (40/983, 4%) were major facilitator superfamily (MFS) transporters (Supplementary Table S8), and 16 of the DEGs (16/983, 1.6%) were ATP-binding cassette (ABC) transporters involved in metabolism (Supplementary Table S9). Some of these transporters might regulate other genes or transport other materials to improve the cordycepin production and consequently be involved in amino acid interconversion and cordycepin metabolism *in vivo*. However, multiple overlapping activities could also promote transporters to interfere in the metabolism of cordycepin and its possible derivatives; further validation of the relation between L-alanine and these transporters remains to be performed in the future.

### Validation of RNA-Seq by qRT-PCR

**FIGURE 6:**
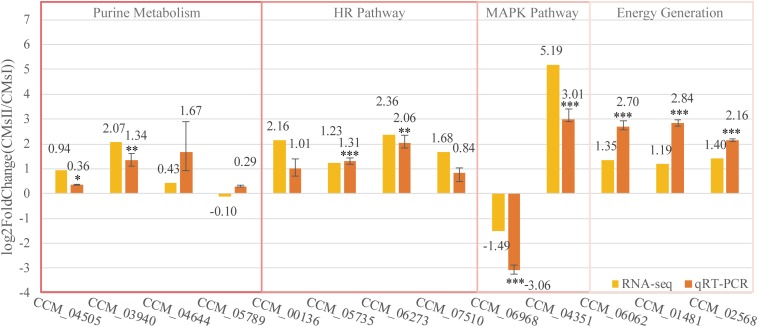
Quantitative real-time PCR validation of DEGs. The qRT-PCR was carried out in biological triplicate. Data were represented as the mean ± SEM (*n* = 9). Statistical analyses were performed using *t-*tests (*p < 0.05, **p < 0.01, and ***p < 0.001). The yellow bars (RNA-seq) were represented in the log2 fold change (CMsII/CMsI) of transcriptome data while the orange bars (qRT-PCR) were represented in the log2 regulation of qRT-PCR data.

To further verify the RNA sequencing data, 13 genes represented four different pathways discussed in this study were selected to perform qRT-PCR validation. The qRT-PCR results (Figure 6 and Supplementary Table S1) showed the differential expression pattern of these genes and further indicated the reliability of RNA-Seq data.

Of these genes, seven genes, which were predicted to participate in cordycepin metabolism as described in Figure 5, CCM_04505 (coding gene of EC 2.4.2.1), CCM_03940 (EC 2.7.4.3), CCM_04644 (EC 3.1.3.5), and CCM_05789 (EC 4.3.2.2), took part in purine metabolism and were responsible for the conversion between ADP, AMP, adenosine, and adenylosuccinate (Figure 5). Though the fold changes of these genes in qRT-PCR were slightly different from the RNA-Seq data, they shared a similar up-regulated tendency. Also, CCM_06062 (annotated as pyruvate kinase), regarded to be involved in energy generation, and CCM_01481 and CCM_02568 (both annotated as Zn2Cys6-type TFs), regarded to play roles in metabolite regulation, have shown significant consistency (*P* < 0.01) of the fold change between qRT-PCR and RNA-Seq results.

Cordycepin, as the analog of adenosine, is capable of blocking the synthesis of DNA or RNA in inflammatory cells. Nevertheless, a high dose of cordycepin showed no obvious effect on mycelial growth in *C. militaris*. The protein Cns3 was also able to synthesize pentostatin, a purine analog that functions as a safeguard of cordycepin to balance its *in vivo* concentration to avoid the toxic effect from a high intracellular level of cordycepin (Xia et al., 2017). The transcriptional level of gene *cns3* showed no difference between groups. Four genes [CCM_00136 (CMsI: 19.2, CMsII: 85.8), CCM_05735 (CMsI: 72.0, CMsII: 169.0), CCM_06273 (CMsI: 9.5, CMsII: 49.1), and CCM_07510 (CMsI: 71.5, CMsII: 228.4)], which were involved in the repair mechanism of the synthesis-dependent strand annealing in the homologous recombination pathway (KEGG ko03440), were significantly up-regulated in RNA sequencing data and the qRT-PCR results. This might indicate that the powerful DNA repair mechanism in *C. militaris* neutralized the toxicity of cordycepin, which causes DNA breaks.

Two other genes, CCM_06968 (Cdc24, CMsI: 53.2, CMsII: 19.0) and CCM_04351 (Flo11, CMsI: 19.4, CMsII: 710.1), which were distinctly different at the transcriptional level and annotated to join in the mitogen-activated protein kinase signaling pathway ko04011, were randomly picked to be verified by qRT-PCR. Their qRT-PCR data were also consistent with the RNA-Seq result.

Overall, these qRT-PCR results showed that the DEGs transcription level was consistent with the data obtained by transcriptome analysis.

### Validation of RNA-Seq by Overexpression of TFs *in vivo*

**FIGURE 7:**
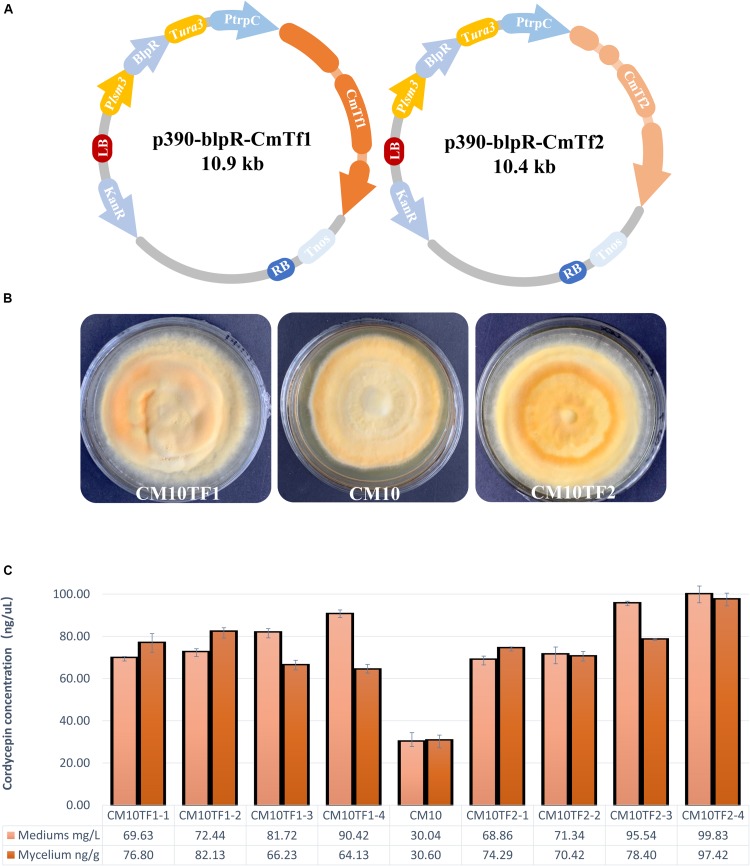
Overexpression of CmTf1 and CmTf2 in *C. militaris*. **(A)** Schematic diagram of CmTf1/2 overexpression vector p390-blpR-CmTf1/2. **(B)** Growth characteristics of *C. militaris* CM10TF1 (left), CM10 (middle), and CM10TF2 (right) on PDA plate. **(C)** Cordycepin production in mediums and mycelium of the *C. militaris* CM10 and CM10TF1/2 after 8 days of flask-shake culturing.

According to the RNA-Seq and qRT-PCR in this study, Zn2Cys6 transcript factors were predicted to be one of the key roles to improve cordycepin production. The most relevant genes in this TF family, which might affect amino acid metabolism or cordycepin production, were CCM_02568 and CCM_01481. To further verify their function, two vectors with the full-length genes under control by a PtrpC promoter were constructed (Figure 7A) to overexpress CmTf1 and CmTf2 individually. These overexpressed fragments of CmTf1/2 were transferred into *C. militaris* genome by ATMT to obtain the CmTf1/2 overexpressed strains and renamed as CM10Tf1/CM10Tf2. The growth rate and pigment accumulation (Figure 7B) showed no obvious difference between the wild-type CM10 and CM10Tf1/CM10Tf2.

Shake-flask fermentation was performed to detect the cordycepin concentration. After culturing for 5 days, L-alanine was added into the fermentation. Mediums and mycelium were collected to perform extraction and detection. As Figure 7C showed, the cordycepin yield of CmTf1/2 overexpressed strains were higher than the wild type. The highest yield of cordycepin is 99 mg/L, which is triple than the wild type in the fermentation medium, while the highest yield is 97 ng/g in the mycelium.

## Discussion

Previous reports (Kang et al., 2014; Zhang et al., 2016) showed that cordycepin production could be increased by adding mineral salts, amino acids, or nucleoside analog. Among these additives, L-alanine was proven to have the best effect on yielding cordycepin (Kang et al., 2014). In this study, to reveal the metabolic pathway of cordycepin, L-alanine was supplemented as an inducer to improve the production of cordycepin in *C. militaris*. After culturing in the liquid static medium for 30 days, the yield of cordycepin was 2.1 times higher than the one without L-alanine supplementary (Figure 1B). Besides, the stage of color change and primordium formation was brought forward by the supplementary. Since cordycepin is a colorless nucleoside analog, the color distinctions in growth characteristics might be due to a higher concentration of pigment accumulation (Figure 1A). Generally, the primordium is formed by gathering a certain amount of mycelium in *C. militaris.* The improved growth rate of mycelia will accumulate more hyphae so that the primordium will generate beforehand. The earlier primordium formation of *C. militaris* indicated that L-alanine might increase cordycepin production by improving the mycelia growth rate.

To figure out the metabolic pathway from the additive L-alanine to cordycepin, four samples were collected and transcriptome sequencing was performed. The transcription data were further elaborated with annotations from the reference database of *C. militaris* and showed that approximately 80% of the genes (Supplementary Table S3) were expressed during the mycelial stage of *C. militaris*. To further verify the data, the read distributions, the Pearson correlation coefficient between expression values across samples, and hierarchical clustering between four samples were calculated (Figures 2A–C). They show which samples shared the same culture condition, which could be considered as reliable biological replicates. The DEG analysis results shown in the volcano plot and heatmap (Figures 2E,F) revealed that adding L-alanine may only induce a few genes to regulate the metabolism *in vivo*, which reveals a possibility to narrow down the DEG targets to figure out which specific genes were taking part in the synthesis and metabolism of cordycepin. To further analyze and to classify the DEGs, the sequencing data were uploaded to the GO and KEGG database (Figure 3). GO classification results of DEGs indicated that the additive might improve the metabolic process to produce more metabolites and consequently activate membrane transportation to avoid unaffordable cordycepin overproduction.

Meanwhile, KEGG classification showed that most DEGs were relevant to the pathway of individual growth and metabolism, which indicated that supplementation with L-alanine might affect the specific amino acid metabolism and energy metabolism. Since amino acids were reported to be involved in the purine pathway (Zheng et al., 2011), to figure out the relationship between L-alanine addition and cordycepin production, metabolic substance transformation was rearranged and showed in a network map. As shown in Figure 4, L-cysteine and L-glutamate were shown as the most important substances while foreign L-alanine was absorbed and included in the metabolism. This indicated that additives like L-alanine may improve cordycepin production by imparting amino acid conversion. Besides, significant changes in the KEGG pathway ko01100 were confirmed as previously mentioned (Vongsangnak et al., 2017). Glycolysis, the pentose phosphate pathway, and the citrate cycle were involved in the biosynthesis of cordycepin by supplying energy for any relevant bioactivities. This indicated that the addition of L-alanine increased energy molecule production and further improved the cordycepin production.

Since few transcription factors were reported to be involved in cordycepin production, the DEGs were uploaded into FIFD and further verified by InterPro to find relevant TF families and genes. The largest TF family was Zn2Cys6, which was also reported as an important part of the fruit body formation of *C. militaris* (Zheng et al., 2011). The most relevant genes in this TF family, which might affect amino acid metabolism or cordycepin production, were CCM_02568 and CCM_01481. Since these genes are highly up-regulated transcripts compared to the treatment, an overexpression was performed in *C. militaris* CM10. The overexpression highly improved the cordycepin production with the addition of L-alanine (Figure 7C) but did not interfere with the mycelia growth rate and pigment accumulation (Figure 7B). This indicated that these transcription factors may not be involved in major physiological activity but only join in specific secondary metabolites to improve cordycepin production. The gene silencing of TFs could further study their function, but the efficient gene inhibition system has not been established in *C. militaris*. Disruption (Chen et al., 2018; Lou et al., 2018) of the genes is another option. The deletion of the transcription factor will lead to multiple effects, because TFs may bind to multiple DNA elements of different genes in different space–time stages. It is hard to build a direct relationship between absent TFs and cordycepin production. Nevertheless, detailed biological functions of CmTf1 and CmTf2 need further researches, such as multiple gene editing or chromatin immunoprecipitation technology. Though evidence was not enough to fulfill the detail of how they regulate the metabolism in this study, they lead to new genome-editing targets for cordycepin overproduction. Moreover, combined with the GO and KEGG results, some genes of the 40 MFS (Supplementary Table S8) and 16 ABC transporters (Supplementary Table S9) might play important roles in amino acid interconversion and cordycepin metabolism *in vivo*.

Even though cordycepin is a purine nucleoside analog, the prediction of the cordycepin pathway in purine metabolism lacked detail. Based on the KEGG ko00230 and cordycepin synthetic pathway in the previous report, results in this study were comprehensively analyzed and the draft of the whole cordycepin metabolic network in *C. militaris* was drawn (Figure 5).

On the one hand, as the KEGG annotation result and the network map shown in Figure 4, L-alanine could improve the cordycepin production by activating the amino acid interconversion. Five amino acids (cysteine, glutamate, glutamine, glycine, and aspartate) showed a clear function in the interconversion and directly stimulated the metabolic substance conversion of histidine metabolism so that it indirectly took part in the cordycepin synthetic pathway.

As a bypass pathway of adenine formation, histidine metabolism was involved in the cordycepin pathway by offering the precursor IMP. Adenylosuccinate synthase (EC 6.3.4.4) gene CCM_06768 could transform IMP into adenylosuccinate, and then the adenylosuccinate lyase (EC 4.3.2.2) gene CCM_05789 will perform the reaction of producing AMP. CCM_06768 was significantly up-regulated while CCM_05789 was not, but the transcriptional level of CCM_05789 was higher than that of 90% DEGs. The gene CCM_00088, annotated as APRTase (EC 2.4.2.7) and conducted the reaction from adenine into AMP, also had a high transcriptional level but only showed slightly up-regulated while compared to the control. The high transcriptional level of these genes indicated that they might be involved in cordycepin synthesis, even though they were not significantly up-regulated in the experimental group with L-alanine addition. Previous studies have noted the importance of adenylate kinase (Kuo et al., 2015) and APRTase (Xiong et al., 2010) in cordycepin synthesis. In this study, adenylate kinase (EC 2.7.4.3) gene CCM_03940 was significantly up-regulated, which might accelerate the conversion from ADP to AMP. This result indicated that the adenylate kinase was one of the rate-limiting enzymes in the cordycepin production pathway.

On the other hand, not only the phenotypic character but also the KEGG classification and ko01100 results indicated that the energy generation was acting an important role in the cordycepin production. From a cellular perspective, pyruvate not only joins in ketone body formation as a precursor but also takes part in ATP generation. In this study, high-level gene expression of pyruvate kinase gene CCM_06062 indicated that it joined in cordycepin metabolism by improving ATP generation. As consistent with the previous report (Mao and Zhong, 2006), H^+^-ATPase genes CCM_01006 and CCM_08873 were highly up-regulated to improve the cordycepin production. This discovery accords with the GO classification and TFs results, suggesting that L-alanine activates additional expression of transporters known to affect energy generation and metabolic substance conversion.

As mentioned above (Lennon and Suhadolnik, 1976), the direct substrate of cordycepin was adenosine. In the purine metabolism, adenosine could be synthesized from three reactions. The first one is conducted by adenosine kinase (EC 2.7.1.20). Although the gene expression level of CCM_02717 did not show significant difference among samples, its transcriptional level was higher than 99.98% DEGs, which indicated that it might be the key enzyme for cell growth rather than the key enzyme in improving cordycepin production by L-alanine. The second pathway was synthesis of adenosine from adenine by purine-nucleoside phosphorylase. However, the transcriptional level of involved gene CCM_04506 (EC 2.4.2.1) was similar to the gene CCM_05789 (EC 4.3.2.2), showing no significant difference between samples. The last one performed a conversion between inosine and adenosine. The enzyme adenosine deaminase was reported to convert adenosine into inosine in the pathway of cytosol RNA editing. Yet, the high transcript gene CCM_07799 (EC 3.5.4.4) might catalyze the reverse reaction to improve the cordycepin production in this study.

Cordycepin was reported to be synthesized from adenosine by a three-gene cassette (Xia et al., 2017). Adenosine is converted to 3′-AMP by Cns3, which also possesses the function of converting adenosine to the cordycepin protector pentostatin. Cordycepin is synthesized after 3′-AMP is converted to the intermediate 2′-carbonyl-3′-deoxyadenosine (2′-C-3′-dA) by the protein complex Cns1-Cns2. In this study, gene *cns1* and *cns2* were significantly up-regulated. This result suggested that the dosage of additive L-alanine in this study was enough to sustain improvement of the cordycepin production. Contrary to expectations, this study showed that gene *cns3* was not significantly up-regulated between samples, indicating that adding L-alanine could not significantly activate the expression of Cns3. One possible hypothesis is that most of substrate 3′-AMP is synthesized from 2′,3′-cAMP through the undetermined enzyme EC 3.1.4.16 during mRNA degradation.

Above all, the combination of transcriptome data in this study and the data from previous reports revealed a reliable metabolic network map of cordycepin production affected by L-alanine addition in the fermentation of *C. militaris*. In brief, the addition of L-alanine increased the cordycepin yield by activating the pathway of energy generation and amino acid interconversion. In the biopathway of energy generation, the activated pyruvate kinase and plasma membrane H^+^-ATPase directly increased the accumulation of ATP, while the Zn2Cys6 TFs (CCM_02568 and CCM_01481) promoted activities in some unknown ways to generate more energy. The consumption of extra ATP could generate a high amount of ADP and AMP, which are precursors of adenosine. In the other biopathway, L-alanine joined in the interconversion of amino acids, the high amount of L-alanine accelerates the formation of amino acids. Through the histidine metabolism, these amino acids indirectly promoted the accumulation of IMP, which is also the precursor of adenosine. The enzymes of the reactions between AMP, IMP, and their intermediate products (adenine, inosine, and adenylosuccinate) to adenosine performed an active stage because of the sufficient AMP and IMP. Thus, the production of cordycepin was increased by excess adenosine and energy.

## Conclusion

We presented the transcriptome analysis of the metabolic network of cordycepin in *C. militaris* activated by L-alanine addition. Up-regulating the transcriptome level of genes involved in the bio-pathways of energy generation and amino acid conversion was the major reason for the overproduction of cordycepin. Two Zn2Cys6-type transcription factors were verified to be related to the rate-limiting steps by increasing the copy number of their coding genes in *C. militaris* wild type. As the whole cordycepin metabolic network map was drawn, this is one step toward discovering the flexibility of cordycepin network and providing a reference for genomic editing to improve cordycepin production as well as other compounds of medicinal value in *C. militaris*.

## Data Availability Statement

The datasets generated for this study can be found in the NCBI Accession: PRJNA413637.

## Author Contributions

B-XC performed data analysis and was a major contributor in drafting the work. TW helped revise the manuscript critically for important intellectual content. B-XC and L-NX performed RNA extraction and qRT-PCR. Q-WZ and Z-WY supervised and B-XC in sequencing data analysis. YZ and YY performed cordycepin detection. FY performed strain culturing. L-QG and J-FL agreed to be accountable for all aspects of the work in ensuring that questions related to the accuracy or integrity of any part of the work are appropriately investigated and resolved. All authors approved the last version of the manuscript to be published.

## Conflict of Interest

FY was employed by Guangzhou Alchemy Biotechnology Co., Ltd. The remaining authors declare that the research was conducted in the absence of any commercial or financial relationships that could be construed as a potential conflict of interest.

## Footnotes

1 http://ftfd.snu.ac.kr/intro.php

2 https://www.ebi.ac.uk/interpro/

## Abbreviations

HPLC: high-performance liquid chromatography
DEGs: differentially expressed genes
FDR: false discovery rate
FPKM: fragments per kilo-base of exon model per million mapped reads
GO: Gene Ontology
KEGG: Kyoto Encyclopedia of Genes and Genomes
TF: transcription factor
ABC: ATP-binding cassette.

## References

[B1] Ashburner M. Ball C. A. Blake J. A. Botstein D. Butler H. Cherry J. M. (2000). Gene Ontology: tool for the unification of biology. *Nat. Genet.* 25 25–29. 10.1038/75556 10802651 PMC3037419

[B2] Audic S. Claverie J. M. (1997). The significance of digital gene expression profiles. *Genome Res.* 7 986–995. 10.1101/gr.7.10.986 9331369

[B3] Chen B.-X. Wei T. Ye Z.-W. Yun F. Kang L.-Z. Tang H.-B. (2018). Efficient CRISPR-Cas9 gene disruption system in edible-medicinal mushroom *Cordyceps militaris*. *Front. Microbiol.* 9:1157 10.3389/fmicb.2018.01157 PMC6005869 29946301

[B4] Cock P. J. A. Fields C. J. Goto N. Heuer M. L. Rice P. M. (2010). The Sanger FASTQ file format for sequences with quality scores, and the Solexa/Illumina FASTQ variants. *Nucleic Acids Res.* 38 1767–1771. 10.1093/NAR/GKP1137 20015970 PMC2847217

[B5] Cohen N. Cohen J. Asatiani M. D. Varshney V. K. Yu H.-T. Yang Y.-C. (2014). Chemical composition and nutritional and medicinal value of fruit bodies and submerged cultured mycelia of culinary-medicinal higher *Basidiomycetes* mushrooms. *Int. J. Med. Mushrooms* 16 273–291. 10.1615/IntJMedMushr.v16.i3.80 24941169

[B6] Cunningham K. G. Manson W. Spring F. S. Hutchinson S. A. (1950). Cordycepin, a metabolic product isolated from cultures of *Cordyceps militaris* (Linn.) link. *Nature* 166 949–949. 10.1038/166949a0 14796634

[B7] Dementhon K. Saupe S. J. Clavé C. (2004). Characterization of IDI-4, a bZIP transcription factor inducing autophagy and cell death in the fungus *Podospora anserina*. *Mol. Microbiol.* 53 1625–1640. 10.1111/j.1365-2958.2004.04235.x 15341644

[B8] Fan D. Wang W. Zhong J.-J. (2012). Enhancement of cordycepin production in submerged cultures of *Cordyceps militaris* by addition of ferrous sulfate. *Biochem. Eng. J.* 60 30–35. 10.1016/j.bej.2011.09.014

[B9] Gajiwala K. S. Burley S. K. (2000). Winged helix proteins. *Curr. Opin. Struct. Biol.* 10 110–116. 10.1016/S0959-440X(99)00057-3 10679470

[B10] Gallegos M. T. Schleif R. Bairoch A. Hofmann K. Ramos J. L. (1997). Arac/XylS family of transcriptional regulators. *Microbiol. Mol. Biol. Rev.* 61 393–410. 9409145 10.1128/mmbr.61.4.393-410.1997 PMC232617

[B11] Gu Y.-X. Wang Z.-S. Li S.-X. Yuan Q.-S. (2007). Effect of multiple factors on accumulation of nucleosides and bases in *Cordyceps militaris*. *Food Chem.* 102 1304–1309. 10.1016/j.foodchem.2006.07.018

[B12] Hall D. B. Wade J. T. Struhl K. (2006). An HMG protein, Hmo1, associates with promoters of many ribosomal protein genes and throughout the rRNA gene locus in *Saccharomyces cerevisiae*. *Mol. Cell. Biol.* 26 3672–3679. 10.1128/MCB.26.9.3672-3679.2006 16612005 PMC1447432

[B13] Jin M. L. Park S. Y. Kim Y. H. Oh J. II Lee S. J. Park G. (2014). The neuroprotective effects of cordycepin inhibit glutamate-induced oxidative and ER stress-associated apoptosis in hippocampal HT22 cells. *Neurotoxicology* 41 102–111. 10.1016/j.neuro.2014.01.005 24486958

[B14] Kanehisa M. Araki M. Goto S. Hattori M. Hirakawa M. Itoh M. (2007). KEGG for linking genomes to life and the environment. *Nucleic Acids Res.* 36 D480–D484. 10.1093/nar/gkm882 18077471 PMC2238879

[B15] Kang C. Wen T.-C. Kang J.-C. Meng Z.-B. Li G.-R. Hyde K. D. (2014). Optimization of Large-Scale culture conditions for the production of cordycepin with *Cordyceps militaris* by liquid static culture. *Sci. World J.* 2014 1–15. 10.1155/2014/510627 PMC4094858 25054182

[B16] Kim D. Langmead B. Salzberg S. L. (2015). HISAT: a fast spliced aligner with low memory requirements. *Nat. Methods* 12 357–360. 10.1038/nmeth.3317 25751142 PMC4655817

[B17] Kim H. G. Shrestha B. Lim S. Y. Yoon D. H. Chang W. C. Shin D.-J. (2006). Cordycepin inhibits lipopolysaccharide-induced inflammation by the suppression of NF-κB through Akt and p38 inhibition in RAW 264.7 macrophage cells. *Eur. J. Pharmacol.* 545 192–199. 10.1016/j.ejphar.2006.06.047 16899239

[B18] Kim S. Park S.-Y. Kim K. S. Rho H.-S. Chi M.-H. Choi J. (2009). Homeobox transcription factors are required for conidiation and appressorium development in the rice blast fungus *Magnaporthe oryzae*. *PLoS Genet.* 5:e1000757 10.1371/journal.pgen.1000757 PMC2779367 19997500

[B19] Kong L. Zhang Y. Ye Z.-Q. Liu X.-Q. Zhao S.-Q. Wei L. (2007). CPC: assess the protein-coding potential of transcripts using sequence features and support vector machine. *Nucleic Acids Res.* 35 W345–W349. 10.1093/nar/gkm391 17631615 PMC1933232

[B20] Kuo H.-C. Huang I.-C. Chen T.-Y. (2015). *Cordyceps s.l.* (*Ascomycetes*) species used as medicinal mushrooms are closely related with higher ability to produce cordycepin. *Int. J. Med. Mushrooms* 17 1077–1085. 10.1615/IntJMedMushrooms.v17.i11.80 26853963

[B21] Langmead B. Salzberg S. L. (2012). Fast gapped-read alignment with Bowtie 2. *Nat. Methods* 9 357–359. 10.1038/nmeth.1923 22388286 PMC3322381

[B22] Le J. Liu J. Bo Z. Feng X. Kexue Y. Fujiang W. (2006). The effect of Zn on the Zn accumulation and biosynthesis of amino acids in mycelia of *Cordyceps sinensis*. *Biol. Trace Elem. Res.* 113 45–52. 10.1385/BTER 17114814

[B23] Lee Y.-R. Noh E.-M. Jeong E.-Y. Yun S.-K. Jeong Y.-J. Kim J.-H. (2009). Cordycepin inhibits UVB-induced matrix metalloproteinase expression by suppressing the NF–κB pathway in human dermal fibroblasts. *Exp. Mol. Med.* 41 548–554. 10.3858/emm.2009.41.8.060 19381070 PMC2739894

[B24] Lennon M. B. Suhadolnik R. J. (1976). Biosynthesis of 3’-deoxyadenosine by *Cordyceps militaris*. *Biochim. Biophys. Acta* 425 532–536. 10.1016/0005-2787(76)90017-4 1083247

[B25] Li B. Dewey C. N. (2011). RSEM: accurate transcript quantification from RNA-Seq data with or without a reference genome. *BMC Bioinformatics* 12:323 10.1186/1471-2105-12-323 PMC3163565 21816040

[B26] Lian T. Yang T. Liu G. Sun J. Dong C. (2014). Reliable reference gene selection for *Cordyceps militaris* gene expression studies under different developmental stages and media. *FEMS Microbiol. Lett.* 356 97–104. 10.1111/1574-6968.12492 24953133

[B27] Livak K. J. Schmittgen T. D. (2001). Analysis of relative gene expression data using real-time quantitative PCR and the 2-ΔΔCT method. *Methods* 25 402–408. 10.1006/meth.2001.1262 11846609

[B28] Lou H. Ye Z. Yun F. Lin J. Guo L. Chen B. (2018). Targeted gene deletion in *Cordyceps militaris* using the split-marker approach. *Mol. Biotechnol.* 60 380–385. 10.1007/s12033-018-0080-9 29605840

[B29] MacPherson S. Larochelle M. Turcotte B. (2006). A fungal family of transcriptional regulators: the zinc cluster proteins. *Microbiol. Mol. Biol. Rev.* 70 583–604. 10.1128/MMBR.00015-06 16959962 PMC1594591

[B30] Maere S. Heymans K. Kuiper M. (2005). BiNGO: a Cytoscape plugin to assess overrepresentation of Gene Ontology categories in Biological Networks. *Bioinformatics* 21 3448–3449. 10.1093/bioinformatics/bti551 15972284

[B31] Mao X.-B. Eksriwong T. Chauvatcharin S. Zhong J.-J. (2005). Optimization of carbon source and carbon/nitrogen ratio for cordycepin production by submerged cultivation of medicinal mushroom *Cordyceps militaris*. *Process Biochem.* 40 1667–1672. 10.1016/j.procbio.2004.06.046

[B32] Mao X.-B. Zhong J.-J. (2006). Significant effect of NH4+ on cordycepin production by submerged cultivation of medicinal mushroom *Cordyceps militaris*. *Enzyme Microb. Technol.* 38 343–350. 10.1016/j.enzmictec.2004.10.010

[B33] Masuda M. Urabe E. Honda H. Sakurai A. Sakakibara M. (2007). Enhanced production of cordycepin by surface culture using the medicinal mushroom *Cordyceps militaris*. *Enzyme Microb. Technol.* 40 1199–1205. 10.1016/J.ENZMICTEC.2006.09.008

[B34] Noh E.-M. Kim J.-S. Hur H. Park B.-H. Song E.-K. Han M.-K. (2008). Cordycepin inhibits IL-1 -induced MMP-1 and MMP-3 expression in rheumatoid arthritis synovial fibroblasts. *Rheumatology* 48 45–48. 10.1093/rheumatology/ken417 19056796

[B35] Park J. Park J. Jang S. Kim S. Kong S. Choi J. (2008). FTFD: an informatics pipeline supporting phylogenomic analysis of fungal transcription factors. *Bioinformatics* 24 1024–1025. 10.1093/bioinformatics/btn058 18304934

[B36] Paterson R. R. M. (2008). *Cordyceps* – A traditional Chinese medicine and another fungal therapeutic biofactory? *Phytochemistry* 69 1469–1495. 10.1016/j.phytochem.2008.01.027 18343466 PMC7111646

[B37] Pertea M. Pertea G. M. Antonescu C. M. Chang T.-C. Mendell J. T. Salzberg S. L. (2015). StringTie enables improved reconstruction of a transcriptome from RNA-seq reads. *Nat. Biotechnol.* 33 290–295. 10.1038/nbt.3122 25690850 PMC4643835

[B38] Raethong N. Laoteng K. Vongsangnak W. (2018). Uncovering global metabolic response to cordycepin production in *Cordyceps militaris* through transcriptome and genome-scale network-driven analysis. *Sci. Rep.* 8 1–13. 10.1038/s41598-018-27534-7 29915355 PMC6006141

[B39] Robinson M. D. McCarthy D. J. Smyth G. K. (2010). edgeR: a Bioconductor package for differential expression analysis of digital gene expression data. *Bioinformatics* 26 139–140. 10.1093/bioinformatics/btp616 19910308 PMC2796818

[B40] Shannon P. Markiel A. Ozier O. Baliga N. S. Wang J. T. Ramage D. (2003). Cytoscape: a software environment for integrated models of biomolecular interaction networks. *Genome Res.* 13 2498–2504. 10.1101/gr.1239303 14597658 PMC403769

[B41] Tarazona S. García-Alcalde F. Dopazo J. Ferrer A. Conesa A. (2011). Differential expression in RNA-seq: a matter of depth. *Genome Res.* 21 2213–2223. 10.1101/gr.124321.111 21903743 PMC3227109

[B42] Taylor M. V. Beatty K. E. Hunter H. K. Baylies M. K. (1995). Drosophila MEF2 is regulated by twist and is expressed in both the primordia and differentiated cells of the embryonic somatic, visceral and heart musculature. *Mech. Dev.* 50 29–41. 10.1016/0925-4773(94)00323-F 7605749

[B43] Tian X. Li Y. Shen Y. Li Q. Wang Q. Feng L. (2015). Apoptosis and inhibition of proliferation of cancer cells induced by cordycepin. *Oncol. Lett.* 10 595–599. 10.3892/ol.2015.3273 26622539 PMC4509066

[B44] Trapnell C. Roberts A. Goff L. Pertea G. Kim D. Kelley D. R. (2012). Differential gene and transcript expression analysis of RNA-seq experiments with TopHat and Cufflinks. *Nat. Protoc.* 7 562–578. 10.1038/nprot.2012.016 22383036 PMC3334321

[B45] Tuli H. S. Sandhu S. S. Sharma A. K. (2014). Pharmacological and therapeutic potential of *Cordyceps* with special reference to Cordycepin. *3 Biotech* 4 1–12. 10.1007/s13205-013-0121-9 PMC3909570 28324458

[B46] Vongsangnak W. Raethong N. Mujchariyakul W. Nguyen N. N. Leong H. W. Laoteng K. (2017). Genome-scale metabolic network of *Cordyceps militaris* useful for comparative analysis of entomopathogenic fungi. *Gene* 626 132–139. 10.1016/j.gene.2017.05.027 28512059

[B47] Xia Y. Luo F. Shang Y. Chen P. Lu Y. Wang C. (2017). Fungal cordycepin biosynthesis is coupled with the production of the safeguard molecule pentostatin. *Cell Chem. Biol.* 24 1–11. 10.1016/j.chembiol.2017.09.001 29056419

[B48] Xiong C. Xia Y. Zheng P. Shi S. Wang C. (2010). TMYC developmental stage-specific gene expression profiling for a medicinal fungus *Cordyceps militaris*. *Mycology* 1 25–66. 10.1080/21501201003674581

[B49] Yin Y. Yu G. Chen Y. Jiang S. Wang M. Jin Y. (2012). Genome-wide transcriptome and proteome analysis on different developmental stages of *Cordyceps militaris*. *PLoS One* 7:e51853 10.1371/journal.pone.0051853 PMC3522581 23251642

[B50] Zhang G. Yin Q. Han T. Zhao Y. Su J. Li M. (2015). Purification and antioxidant effect of novel fungal polysaccharides from the stroma of *Cordyceps kyushuensis*. *Ind. Crops Prod.* 69 485–491. 10.1016/j.indcrop.2015.03.006

[B51] Zhang Q. Liu Y. Di Z. Han C. Liu Z. (2016). The strategies for increasing cordycepin production of *Cordyceps militaris* by liquid fermentation. *Fungal Genomics Biol.* 6 1–5. 10.4172/2165-8056.1000134

[B52] Zheng P. Xia Y. Xiao G. Xiong C. Hu X. Zhang S. (2011). Genome sequence of the insect pathogenic fungus *Cordyceps militaris*, a valued traditional chinese medicine. *Genome Biol.* 12:R116 10.1186/gb-2011-12-11-r116 PMC3334602 22112802

[B53] Zhou X. Cai G. He Y. Tong G. (2016). Separation of cordycepin from *Cordyceps militaris* fermentation supernatant using preparative HPLC and evaluation of its antibacterial activity as an NAD+-dependent DNA ligase inhibitor. *Exp. Ther. Med.* 12 1812–1816. 10.3892/etm.2016.3536 27588098 PMC4998010

[B54] Zhou X. Gong Z. Su Y. Lin J. Tang K. (2009). *Cordyceps* fungi: natural products, pharmacological functions and developmental products. *J. Pharm. Pharmacol.* 61 279–291. 10.1211/jpp.61.03.0002 19222900

